# Efficacy and safety of endoscopic ultrasonography‐guided radiofrequency ablation of small pancreatic neuroendocrine neoplasms: A prospective, pilot study

**DOI:** 10.1002/deo2.70073

**Published:** 2025-01-29

**Authors:** Kazuyuki Matsumoto, Daisuke Uchida, Yasuto Takeuchi, Hironari Kato, Yuki Fujii, Kei Harada, Nao Hattori, Ryosuke Sato, Taisuke Obata, Akihiro Matsumi, Kazuya Miyamoto, Shigeru Horiguchi, Koichiro Tsutsumi, Kazuya Yasui, Ryo Harada, Masakuni Fujii, Motoyuki Otsuka

**Affiliations:** ^1^ Department of Gastroenterology and Hepatology Okayama University Hospital Okayama Japan; ^2^ Department of Gastroenterological Surgery, Transplant and Surgical Oncology Okayama University Hospital Okayama Japan; ^3^ Department of Gastroenterology Japanese Red Cross Okayama Hospital Okayama Japan; ^4^ Department of Internal Medicine Okayama Saiseikai General Hospital Okayama Japan

**Keywords:** ablation techniques, endosonography, neuroendocrine tumors, pancreatic neoplasms, pilot projects

## Abstract

**Objectives:**

Endoscopic ultrasonography (EUS)‐guided radiofrequency ablation has recently been introduced as one of the management strategies for small pancreatic neuroendocrine neoplasms (PNENs). However, prospective data on its safety and efficacy remain limited.

**Methods:**

This prospective pilot study was conducted at Okayama University Hospital from May 2023 to December 2024. Patients with grade 1 PNENs ≤15 mm, confirmed by EUS‐guided fine‐needle aspiration, were included. The primary endpoint was safety (adverse events [AEs] evaluated according to the 2010 guidelines of the American Society for Gastrointestinal Endoscopy. Severe AEs were defined as moderate or higher in American Society for Gastrointestinal Endoscopy grading and grade ≥3. Secondary endpoints included efficacy (complete response on contrast‐enhanced computed tomography at 1 and 6 months), treatment details, device failure, diabetes mellitus exacerbation, and overall survival at 6 months.

**Results:**

Five patients with non‐functional PNENs (median age: 64 years; median tumor size: 10 mm) were treated. AEs occurred in two patients (40%, 2/5), although none was severe. Both patients developed asymptomatic pseudocysts, one experienced mild pancreatitis, and both resolved with conservative treatment. The complete response rates on contrast‐enhanced computed tomography at one and 6 months were 100%. The median procedure time was 16 min without any device failure, and the median hospitalization was 5 days. None of the patients developed new‐onset or worsening diabetes mellitus. The 6‐month overall survival rate was 100%.

**Conclusion:**

EUS‐guided radiofrequency ablation demonstrated a high complete response rate with no severe AEs in this pilot study, suggesting a minimally invasive option for small, low‐grade PNENs (jRCTs062230014).

## INTRODUCTION

Pancreatic neuroendocrine neoplasms (PNENs) are rare, representing only 1%–2% of all primary pancreatic cancers.^1^ However, their detection has increased significantly due to the widespread use of advanced endoscopic and radiological imaging techniques.^2^

The choice of treatment for PNENs depends on factors such as the presence of hormone‐related symptoms and the tumor size.^3^, ^4^ Surgical resection is typically recommended for patients with symptomatic tumors or those with tumors > 2 cm in diameter. However, the optimal treatment approach for small, non‐functional, low‐grade PNENs (≤ 2 cm) remains a subject of ongoing debate. Pancreatic surgery is associated with a higher risk of complications than other gastrointestinal surgeries. Additionally, it can lead to reduced pancreatic endocrine and exocrine function following resection. Therefore, the decision to proceed with surgery must carefully weigh the potential benefits against the risk of postoperative complications.^3^, ^4^, ^5^

For PNENs < 2 cm in size with low‐grade malignancy, a watch‐and‐wait strategy is commonly adopted.^3^, ^4^ A recent study on patients with small, non‐functional PNENs who underwent surgical resection found no significant difference in the 5‐year cancer‐specific survival compared to those managed through observation.^4^ However, long‐term outcomes beyond 5 years and the characteristics of small tumors that are likely to progress remain unclear. Follow‐up typically involves annual imaging with contrast agents, which presents challenges such as the risk of allergic reactions to contrast agents, potential kidney dysfunction, cumulative lifetime radiation exposure, and high medical costs. Additionally, surgery is recommended if the tumor grows beyond 2 cm. Given these limitations, there is an urgent need for minimally invasive treatment options as alternatives to surgery for low‐grade small PNENs.

Recent advances in endoscopic ultrasound (EUS)‐guided ablative techniques have provided a possible alternative to surgical resection. The advantages of EUS‐guided local ablation include reduced complications and preserved pancreatic function. EUS‐guided radiofrequency ablation (RFA) is one of the most promising treatment options for pancreatic tumors, especially for PNENs, as RFA induces thermal necrosis of the tumor mass.^6^, ^7^, ^8^, ^9^, ^10^, ^11^, ^12^, ^13^, ^14^ However, prospective studies on EUS‐RFA for PNENs are limited, and further investigation is required to evaluate its safety and efficacy.^7^, ^8^, ^9^, ^10^ Therefore, we conducted a prospective pilot study to evaluate the safety and efficacy of EUS‐RFA for small, low‐grade PNENs.

## METHODS

### Study design and participants

**FIGURE 1:**
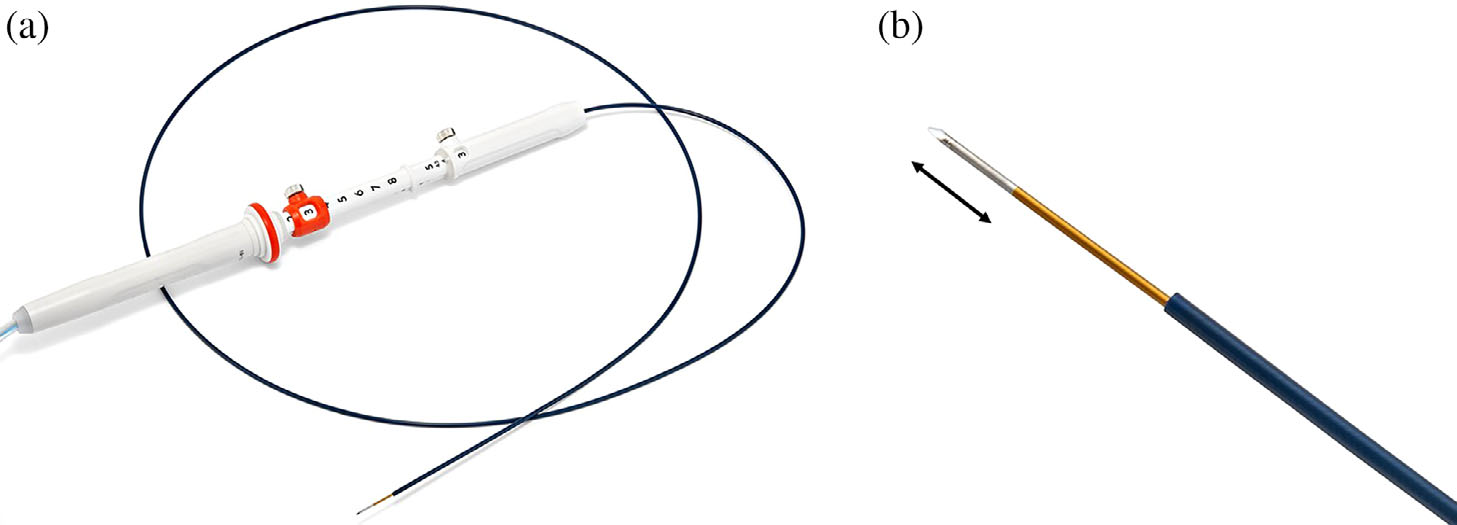
(a): EUSRA RF electrode (19 G; STARmed, Taewoong, Korea). (b): Tip of the needle. Ablation zone (double arrow)

A prospective pilot study was conducted at Okayama University Hospital from May 2023 to December 2024. Figure 1 shows the flow diagram of patient enrollment and an overview of the study protocol. The eligibility criteria included: (1) age ≥ 18 years, (2) provision of informed consent, (3) grade 1 PNEN diagnosed pathologically using EUS‐guided fine‐needle aspiration (FNA) specimens (World Health Organization 2017 classification), (4) a tumor diameter ≤15 mm on contrast‐enhanced (CE)‐CT, and (5) PNEN diagnosed as a non‐functional tumor or insulinoma. The exclusion criteria included: (1) allergy to contrast media, (2) presence of a cardiac pacemaker, (3) distance between the tumor and main pancreatic duct of ≤ 2 mm, (4) suspicion of lymph node metastasis or distant metastasis on CE‐CT, (5) prothrombin time less than 50% or an international normalized ratio greater than 1.5, (6) platelet count less than 50,000/µL, (7) estimated glomerular filtration rate less than 30 mL/min/1.73 m^2^, (8) use of more than two antithrombotic agents, (9) performance status of 2 or higher, (10) pregnancy or suspected pregnancy, and (11) life expectancy of less than 5 years.

This study was approved by the Ethical Review Board of Okayama University Hospital (approval number CRB23‐001). The protocol was registered in the Japan Registry of Clinical Trials (clinical research protocol number: jRCTs062230014). Written informed consent was obtained from all patients before inclusion. This study was conducted in accordance with the principles of the Declaration of Helsinki. We also established an independent data monitoring committee comprising three additional doctors (Ryo Harada, Kazuya Yasui, and Masakuni Fujii) who were not associated with the study to determine whether the study should continue if severe adverse events (AEs) occurred.

### Endpoints

The primary endpoint of the study was safety. Procedure‐related AEs were evaluated based on the 2010 guideline of the American Society for Gastrointestinal Endoscopy (ASGE).^15^ Severe AEs were defined as moderate or higher in ASGE and grade ≥3. Complications were evaluated based on the timing of onset and classified as early (less than 4 weeks) or late (4 weeks or more).

The following secondary endpoints were evaluated: (1) Efficacy: Complete response on CE‐CT 1 and 6 months after treatment. (2) Evaluation of procedural details and treatment outcomes. (3) Device failure. (4) New onset diabetes mellitus (DM) or DM exacerbation 6 months after treatment. (5) Six months overall survival. Complete response was defined as the absence of enhanced areas within the tumor on arterial‐phase CE‐CT images with a 1–2‐mm thick slice. Two expert gastroenterologists independently reviewed the CE‐CT images based on the radiologist's findings. If a judgment could not be made using CE‐CT, CE‐EUS with perflubutane (Daiichi‐Sankyo Co., Ltd.) was performed to assess the enhanced areas within the tumor. In this pilot study, evaluation of recurrence around the lesion or distant metastasis was not conducted, and the outcome was focused on the assessment of the ablation area. DM was defined as fasting or non‐fasting blood glucose levels of ≥126 or 200 mg/dL, respectively, and glycated hemoglobin levels of ≥ 6.5 (National Glycohemoglobin Standardization Program value). New‐onset DM was defined as DM presence 6 months after treatment despite DM absence at the time of registration. Exacerbation was defined as the need to start, add, or increase the dosage of DM medications due to poor glycemic control in patients with DM at the time of registration. Patients with clear exacerbating factors, such as weight gain or overeating, were not considered to have new‐onset or worsening DM. If judgment was difficult, an evaluation was requested by an independent data‐monitoring committee. The evaluation was conducted 6 months post‐treatment.

### Study procedure

The procedure was performed with patients in the prone or semi‐prone position under conscious sedation in an endoscopy room using intravenous anesthetic. Before the procedure, a 50‐mg diclofenac suppository was used to prevent pancreatitis. Antibiotic prophylaxis (sulbactam/cefoperazone 1 g two times per day intravenously for 3 days) was used to prevent infection. EUS‐RFA was performed by an experienced endosonographer with over 10 years of experience in EUS‐related procedures using a EUS therapeutic scope (UCT260; Olympus). A 19‐gauge RFA needle (STARmed) was used (Figure 1), and the ablation protocol was based on the manufacturer's instructions (Table S1). The delivering ablation power of 10–30 W in continuous mode was connected to a dedicated radiofrequency current generator (VIVA RF generator). The needle size was selected based on the diameter of the tumor, with reference to the ablation volume data provided by the manufacturer (Table S2). When a hypoechoic area clearly remained after ablation, additional punctures and ablations were performed at the site. The needle was left in the tumor for approximately one minute after ablation before being withdrawn. The RFA procedure was terminated when the impedance exceeded 4–500 Ω (Video S1). The RFA needle was equipped with an internal cooling system to minimize tissue heat damage. CE‐CT was performed 3 days after the procedure to evaluate tumor viability and procedure‐related AEs. If there were no issues with CT findings, physical examination, or blood tests, the patient was discharged the following day. If any abnormalities were detected, hospitalization was extended until improvement was observed.

### Follow‐up

Patients were followed for 6 months to assess the acute and sub‐acute post‐treatment course. Follow‐up examinations were scheduled at 1, 3, and 6 months to evaluate the patient's general condition and to perform blood tests. The patients were scheduled to undergo follow‐up CE‐CT imaging 1 and 6 months after discharge. Under the protocol, salvage surgical resection was to be recommended when follow‐up CE‐CT showed signs of incomplete response.

### Sample size calculation

This study was exploratory, with the sample size determined based on the outpatient population. The selected case count reflects the number of feasible cases that could be achieved within the designated study period. Therefore, five cases were included as examples of the investigational treatment.

### Statistical analysis

The analysis population included all participants who were registered in the study and underwent procedures according to the protocol. Continuous variables were reported as ranges, and categorical variables as counts and percentages. All analyses were performed using JMP Pro 17 software (SAS Institute, Cary, NC, USA).

## RESULTS

### Study population

**FIGURE 2:**
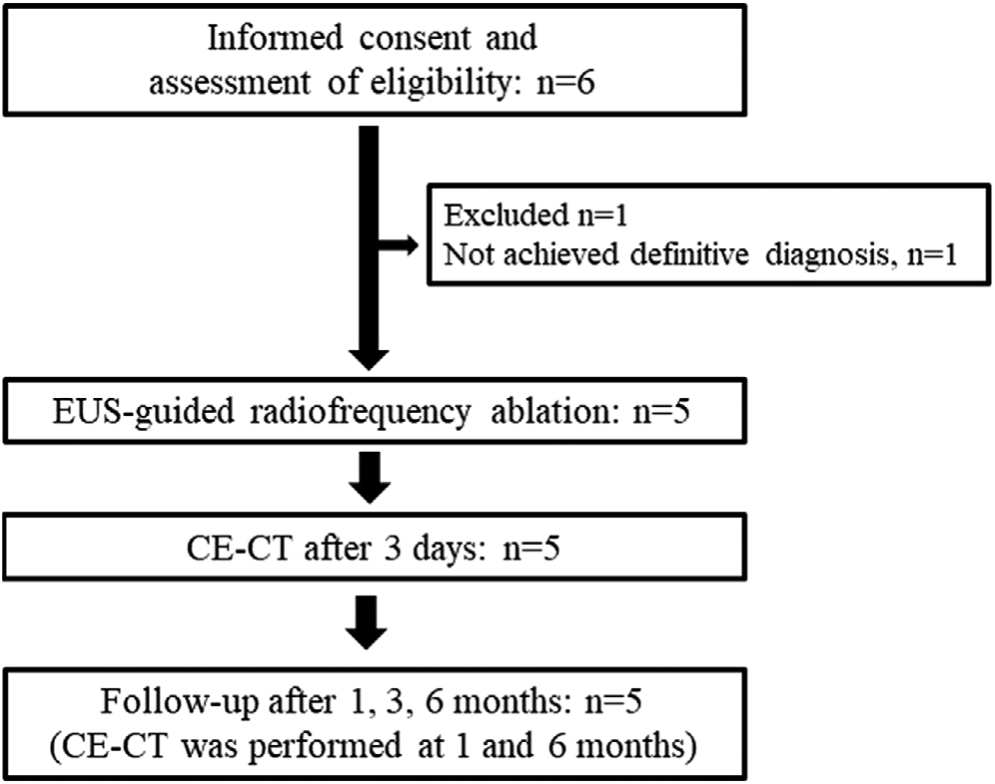
Flowchart of the study. CE‐CT, contrast‐enhanced computed tomography; EUS, endoscopic ultrasonography

Of the six eligible patients who provided informed consent, one was excluded (no definitive diagnosis of PNEN using EUS‐FNA). Overall, five patients were analyzed and treated with EUS‐RFA. After treatment, all patients completed the scheduled follow‐up protocol (Figure 2). The characteristics and treatment outcomes of the enrolled patients are shown in Table 1. The median age of the patients was 64 years (range: 46–71). The median tumor size was 10 mm (range: 7–12). The locations of the tumors were as follows: head (one patient), body (two patients), and tail (two patients). The median distance between the tumor and the main pancreatic duct was 2.5 mm (range 2.0–2.5). All patients had non‐functional tumors.

**TABLE 1:** Patient characteristics and treatment outcomes of endoscopic ultrasound‐guided radiofrequency ablation.

												Adverse events			
Patient no.	Age, years	Sex	Location	Size, mm	Distance between tumor and MDP, mm	Needle size, mm	Power, Wattage	Total ablation time, s	No. of ablations/ session	Procedure time, min	Hospitali zation days	Early	Late	Complete response at 1 and 6 months	Development of DM
1	64	M	Pt	10	5	7	20	20	1	10	5	None	None	Success	None
2	65	F	Pb	7	2.2	5	10	25	1	16	5	None	None	Success	None
3	58	M	Ph	12	2.5	7	20	97	3	21	5	None	Pseudocyst	Success	None
4	71	F	Ph	10	5	7	20	40	1	9	5	None	None	Success	None
5	46	M	Pt	10	3	10	30	67	3	20	7	Pancreatitis	Pseudocyst	Success	None

*Note*: M: male, F: female, Ph: pancreatic head, Pb: pancreatic body, Pt: pancreatic tail, MDP: main pancreatic duct, DM: diabetes mellitus.

### Primary endpoint

**FIGURE 3:**
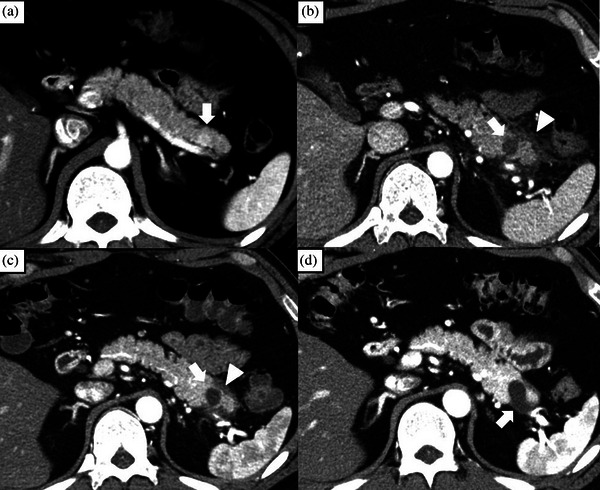
Patients with acute pancreatitis after EUS‐guided radiofrequency ablation (Patient number 5). (a): An enhanced tumor measuring 10 mm located in the tail of the pancreas on CE‐CT (arrow). (b): CE‐CT performed 3 days after the procedure revealed that the tumor site was ablated (arrow) and inflammatory findings had spread beyond the pancreas (arrowhead). (c): CE‐CT scan at 1 month showed that the enhancement of the tumor had disappeared (arrow), and the peripancreatic inflammation had almost resolved (arrowhead). (d): CE‐CT at 6 months reveals the formation of a pseudocyst at the ablation site (arrow). CE‐CT, contrast enhanced‐computed tomography; EUS, endoscopic ultrasonography.

**FIGURE 4:**
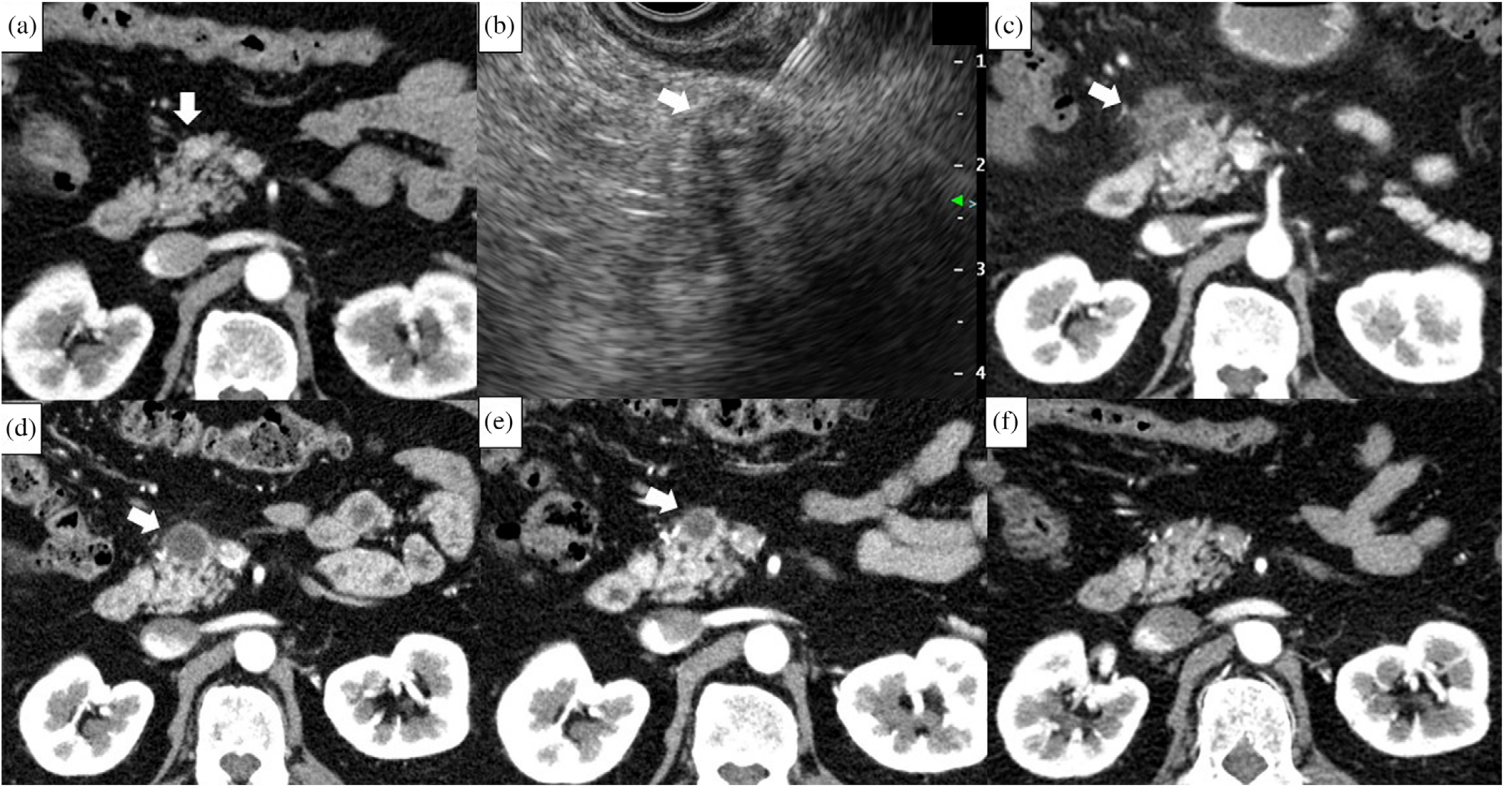
Patient with pseudocyst after EUS‐guided radiofrequency ablation (Patient number 3). (a): An enhanced tumor measuring 12 mm is located at the head of the pancreas on CE‐CT (arrow). (b): A 19‐gauge needle was inserted into the tumor, and EUS‐guided ablation was performed (arrow). (c): CE‐CT performed 3 days after the procedure revealed that the enhancement of the tumor had completely disappeared; however, inflammatory findings had spread beyond the pancreas (arrow). (d): CE‐CT at 1 month reveals the formation of a pseudocyst at the ablation site (arrow). (e): The pseudocyst has decreased in size on CE‐CT at 6 months; however, it is still present (arrow). (f): The pseudocyst has disappeared on CE‐CT at one year. CE‐CT, contrast enhanced‐computed tomography; EUS: endoscopic ultrasonography.

The prevalence of AEs was 40% (2/5). No severe AEs occurred in any patient. Early AE occurred in one patient with mild pancreatitis (Figure 3). Late AEs occurred in two cases, both of which were asymptomatic pseudocysts that resolved spontaneously without therapeutic interventions (Figure 4).

### Secondary endpoints

**FIGURE 5:**
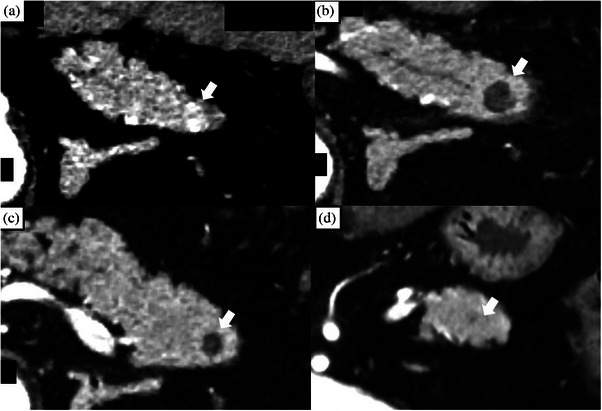
Patients without complications after EUS‐guided radiofrequency ablation (Patient number 1). (a): An enhanced tumor measuring 10 mm located in the tail of the pancreas on CE‐CT (arrow). (b): CE‐CT performed 3 days after the procedure reveals that the tumor site is ablated (arrow). (c): CE‐CT at 1 month showing that the enhancement of the tumor had disappeared (arrow) and the ablated site was gradually absorbed. (d): The ablation site is barely detectable on CE‐CT at 6 months (arrow). CE‐CT, contrast‐enhanced computed tomography; EUS, endoscopic ultrasonography.

**FIGURE 6:**
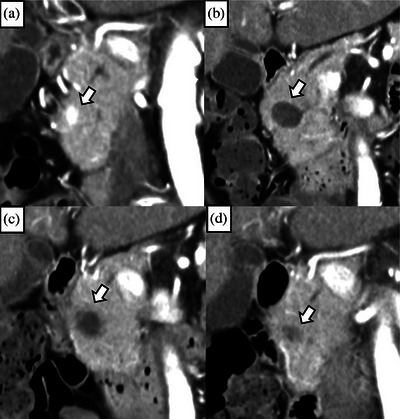
Patients without complications after EUS‐guided radiofrequency ablation (Patient number 2). (a): An enhanced tumor measuring 7 mm located in the body of the pancreas on CE‐CT (arrow). (b): CE‐CT performed 3 days after the procedure reveals that the tumor site is ablated (arrow). (c): CE‐CT at 1 month showing that the enhancement of the tumor had disappeared (arrow) and the ablated site was gradually absorbed. (d): The ablation site is shrinking on CE‐CT at 6 months (arrow). CE‐CT, contrast‐enhanced computed tomography; EUS, endoscopic ultrasonography.

**FIGURE 7:**
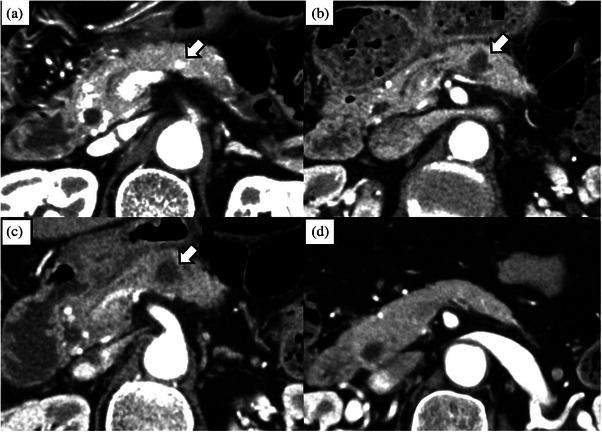
Patients without complications after EUS‐guided radiofrequency ablation (Patient number 4). (a): An enhanced tumor measuring 10 mm located in the head of the pancreas on CE‐CT (arrow). (b): CE‐CT performed 3 days after the procedure reveals that the tumor site is ablated (arrow). (c): CE‐CT at 1 month showing that the enhancement of the tumor had disappeared (arrow) and the ablated site was still present. (d): The ablation site cannot be identified on CE‐CT at 6 months. CE‐CT, contrast‐enhanced computed tomography; EUS, endoscopic ultrasonography.

The complete response rates on CE‐CT at 1 and 6 months were 100% (5/5). The 6‐month overall survival rate was 100% (5/5). The needles used were 5 mm in one patient, 7 mm in three patients, and 10 mm in one patient. The power settings used were 10 W in one patient, 20 W in three patients, and 30 W in one patient. The number of ablations per session was once in three patients and three times in two patients. The median procedure time was 16 min (range, 9–21 min), and the ablation time was 30 s (range: 20–97 s). The median hospitalization duration was 5 days (range: 5–7 days). DM incidence or its exacerbation at 6 months was 0% (0/5). None of the patients required further surgery. In cases without complications, the ablation site was gradually absorbed and became almost indistinct on CT at 6 months (Figures 5, 6, 7).

## DISCUSSION

This prospective pilot study evaluated EUS‐RFA for small grade 1 PNENs. Evaluations were performed according to the recommendations of the product manual in this pilot study. Although no severe AEs were observed, 40% (2/5) of patients experienced complications, both of which were associated with prolonged ablation time. The complete response rate was 100%, demonstrating the effectiveness of the treatment. Although review articles on EUS‐RFA outcomes for PNENs include approximately 300 cases,^6^ only four articles that involved 37 cases have been studied in prospective research.^7^, ^8^, ^9^, ^10^ Furthermore, there are no reports detailing the protocols for ablation wattage or duration, highlighting the need for further studies on ablation parameters.

Khoury et al.’s^6^ meta‐analysis of EUS‐RFA for locoregional PNENs reported a technical success rate of 99.2% (95% confidence interval [CI], 97.9%–99.9%), complete radiological response of 87.1% (95% CI, 80.1%–92.8%), and an AE incidence of 20.0% (95% CI, 14.0%–26.7%), whereas the incidence of severe AEs was 0.9% (95% CI 0.2%–2.3%). The most common AEs were transient mild abdominal pain (19 patients, 6.5%) and mild‐to‐moderate pancreatitis (23 patients, 7.9%). Although no severe AEs were observed, AEs incidence in our pilot study was high, which is believed to be dependent on the wattage used and the timing of ablation completion. Interestingly, in their report, complete response was associated with the power settings of the RFA system: a power setting of <50 W achieved a complete response in 92.4% of the cases, whereas 50 W achieved a complete response in 84.6%. In RFA treatment, using a lower wattage for ablation results in longer ablation times and broader ablation areas than using a higher wattage.^16^ In this pilot study, the procedure was performed using the recommended wattage according to the needle size, and ablation was basically continued until impedance rose sufficiently (4–500 Ω). As a result, the median total ablation time was prolonged to 40 s, and complications occurred in two patients that required three ablation sessions (total ablation times of 67 and 97 s). In this study, all patients achieved complete response. Other studies have not clearly specified the appropriate endpoint for ablation, whether it should be when the bubbles reach the tumor margin or when the impedance rises sufficiently. Prolonged ablation times are expected to increase the extent of ablation and cause greater damage to pancreatic tissue. Further investigations are required to determine the optimal ablation protocol.

Recently, EUS‐guided ethanol injection (EUS‐EI) and EUS‐RFA have been performed for small PNENs. A recent meta‐analysis including 181 patients (100 EUS‑RFA, 81 EUS‐EI) with PNENs (mean size 15.1 ± 4.7 mm) reported no significant difference in the rates of technical success (94.4% vs. 96.7%, *p *= 0.42), clinical success (85.2 vs. 82.2%, *p *= 0.65), and AEs (14.1% vs. 11.5%, *p *= 0.7) between EUS‑RFA and EUS‑EI, respectively.^17^ However, the included reports studied only non‐functional PNENs, and the complete response rate for EUS‐EI (60%–80%) was lower than that for EUS‐RFA (86%–100%).^18^ Matsumoto et al.^19^ conducted a prospective multicenter study of EUS‐EI in 25 patients with PNENs (G1) measuring 15 mm or less and reported a complete response rate of 88% at 6 months. However, based on tumor size, the complete response rates were 91.7% (11/12) and 84.6% (11/13) for tumors <10 and 10–15 mm, respectively. So et al.’s study on long‐term treatment outcomes of EUS‐EI for small PNENs revealed that of the 97 patients treated with EUS‐EI, 63 (65%) showed complete response (mean tumor size, 12.08 ± 3.6 mm).^20^ Although the results were obtained in a relatively small number of cases, EUS‐EI may be sufficiently effective, particularly for tumors <10 mm in size. In this pilot study, the tumor size was limited to ≤ 15 mm to prioritize safety. However, tumors measuring 10–20 mm are considered good candidates for EUS‐RFA.

EUS‐RFA is anticipated to be a minimally invasive alternative surgical treatment for PNEN localized in the pancreas. According to a recent large‐scale retrospective study that used propensity‐matching analysis to compare the outcomes of EUS‐RFA and surgery for pancreatic insulinoma,^21^ there was no significant difference in clinical efficacy defined as the complete resolution of insulin‐related symptoms between surgery and EUS‐RFA (100% [89/89] vs. 95.5% [85/89], *p *= 0.160). However, the rate of AEs was significantly lower for EUS‐RFA at 18.0% (16/89) than for surgery at 61.8 % (55/89; *p *< 0.001). Severe AEs occurred in 15.8% (14/89) of surgical cases but were not observed in any EUS‐RFA cases (0%; *p *< 0.0001). The study concluded that EUS‐RFA is safer than surgery and is highly effective in treating pancreatic insulinoma. During the median follow‐up period of 23 months, 15 patients (16.9%) experienced symptom recurrence after undergoing EUS‐RFA. Therefore, further research is required to assess the long‐term outcomes of EUS‐RFA. In terms of pancreatic function, procedures like pancreaticoduodenectomy and distal pancreatectomy, which involve extensive pancreatic resection, lead to postoperative complications such as DM and impaired nutrient absorption in 14%–18% and 17–33% of cases, respectively.^5^, ^22^ In this pilot study, none of the patients developed new‐onset DM or experienced worsening of existing DM within 6 months after EUS‐RFA. The treatment also preserved pancreatic function.

In conclusion, EUS‐RFA appears to be a safe, effective, and minimally invasive technique for the treatment of small PNENs. However, the prolonged ablation time due to the ablation protocol resulted in minor complications. Further investigations are needed to determine the optimal ablation protocol to reduce AEs and control the ablation area.

## CONFLICT OF INTEREST STATEMENT

This study received a free supply of the EUSRA RF Electrode and VIVA Combo RF System from TaeWoong Medical.

## ETHICS STATEMENT

The Institutional Review Board of our hospital approved this study (Approval Number: CRB23‐001).

## PATIENT CONSENT STATEMENT

We obtained written informed consent from all patients.

## CLINICAL TRIAL REGISTRATION

The protocol was registered in the Japan Registry of Clinical Trials (clinical research protocol number: jRCTs062230014).

## Supporting information



TABLE S1 Protocol for endoscopic ultrasound‐guided radiofrequency ablation.
TABLE S2 Ablation volume with 19‐gauge RFA needle.

VIDEO S1 EUS‐guided radiofrequency ablation (Patient number 4).

## Data Availability

The datasets used and/or analyzed during the current study are available from the corresponding author upon reasonable request.
