# Supplementary material for: Efficacy and safety of endoscopic ultrasonography‐guided radiofrequency ablation of small pancreatic neuroendocrine neoplasms: A prospective, pilot study
Source: DEN Open. 2025 Jan 29;5(1):e70073. doi: 10.1002/deo2.70073 (PMC11779739; doi:10.1002/deo2.70073)
Supplement: Supplementary file 1 — TABLE S1 Protocol for endoscopic ultrasound‐guided radiofrequency ablation. TABLE S2 Ablation volume with 19‐gauge RFA needle. [file DEO2-5-e70073-s001.docx]

**Supplementary Material**

**Author Names**

Kazuyuki Matsumoto, Daisuke Uchida, Yasuto Takeuchi, Hironari Kato, Yuki Fujii, Kei Harada, Nao Hattori, Ryosuke Sato, Taisuke Obata, Akihiro Matsumi, Kazuya Miyamoto, Shigeru Horiguchi, Koichiro Tsutsumi, Kazuya Yasui, Ryo Harada, Masakuni Fujii and Motoyuki Otsuka

**Title of Paper**

Efficacy and Safety of Endoscopic Ultrasonography-Guided Radiofrequency Ablation of Small Pancreatic Neuroendocrine Neoplasms: A Prospective, Pilot Study

| Supplemental Table 1. Protocol for endoscopic ultrasound-guided radiofrequency ablation | | |  |
| --- | --- | --- | --- |
|  |  |  |  |
| Needle size, mm | Power, Wattage | Duration (when to stop) |  |
|  |  |  |  |
| 5 | 10 | Until echogenic bubble is visible along the whole body or the needle or Until impedance rise enough (above 4-500 ohm.) |  |
| 7 | 20 |  |  |
| 10 | 30-50 |  |  |
| 15 or 20 | 50 |  |  |
| Recommendation of the manufacturer | | |  |

| Supplemental Table 2. Ablation volume with 19-Gauge RFA needle | | | |  |
| --- | --- | --- | --- | --- |
|  | | | |  |
| Bovine ex-vivo test | | | |  |
| Needle size, mm | Wattage | Ablation time, sec | Width×length, mm |  |
|  |  |  |  |  |
| 5 | 10 | 10 | 5.6 × 6.9 |  |
| 7 | 20 | 15 | 7.6 × 9.0 |  |
| 10 | 30 | 20 | 8.6 × 14.0 |  |
| Data from the manufacturer | | | |  |

**Video legend**

Video title: EUS-guided radiofrequency ablation (Patient number 4)

1. There is a tumor with 10mm diameter in the head of the pancreas.

2. A 19-gauge needle with 7mm tip was inserted into the tumor.

3. Ablation was started and bubbles spread within the tumor before the impedance rises enough.

4. The ablation was terminated when the impedance exceeded 4-500 Ohms (Ω).

5. No bleeding points were observed with the color Doppler image.
